# Preparation and novel photoluminescence properties of the self-supporting nanoporous InP thin films

**DOI:** 10.1038/s41598-020-77651-5

**Published:** 2020-11-25

**Authors:** Dezhong Cao, Bo Wang, Dingze Lu, Xiaowei Zhou, Xiaohua Ma

**Affiliations:** 1 School of Science, Xi’an Polytechnic University, Xi’an, 710048 People’s Republic of China; 2 Key Laboratory of Ministry of Education for Wide Band-Gap Semiconductor Materials and Devices, School of Microelectronic, Xidian University, Xi’an, 710126 People’s Republic of China

**Keywords:** Nanoscience and technology, Optics and photonics

## Abstract

Self-supporting nanoporous InP membranes are prepared by electrochemical etching, and are then first transferred to highly reflective (> 96%) mesoporous GaN (MP-GaN) distributed Bragg reflector (DBR) or quartz substrate. By the modulation of bandgap, the nanoporous InP samples show a strong photoluminescence (PL) peak at 541.2 nm due to the quantum size effect of the nanoporous InP structure. Compared to the nanoporous InP membrane with quartz substrate, the nanoporous membrane transferred to DBR shows a twofold enhancement in PL intensity owing to the high light reflection effect of bottom DBR.

## Introduction

In recent years, III-V semiconductors have been widely used in microelectronics and optoelectronic devices^1–8^. In particularly, indium phosphide (InP), which is a well-known direct bandgap semiconductor, has attracted great attention in optoelectronic devices^1,2,7^. Nevertheless, high costs and narrow bandgap (E_g_ = 1.34 eV) (in the near infrared region) limits its applications in the visible range.

To solve the above problems, the lift-off of InP membranes from bulk InP has attracted increasing attention. One of the approaches is the SmartCut method. The self-supporting InP membrane can be prepared with (hydrogen or helium) implantation-induced cracks^9^. An alternative method is electrochemical etching which has been applied widely to prepare the lift-off porous Si thin films^10^ and self-supporting nanoporous GaN thin films^11,12^. More interestingly, the PL peak positions and intensity of porous Si and InGaN-based films can be modulated via electrochemical etching^13,14^, which makes the modulation of bandgap and PL intensity of InP possible. Additionally, distributed Bragg reflector (DBR) plays an important role in the development of optoelectronic devices^15,16^. Supposing that the lift-off nanoporous InP membrane with visible PL is transferred to a DBR substrate, the PL emission can be further enhanced. However, no report focuses on them.

Herein, lift-off nanoporous InP membranes with visible PL are prepared by an electrochemical etching, and then are transferred to MP-GaN DBR or quartz substrates for the first time. Compared to the InP membrane with quartz substrate, the membrane transferred to DBR substrate shows greater PL efficiency in the visible range.

## Results and discussion

**Figure 1:**
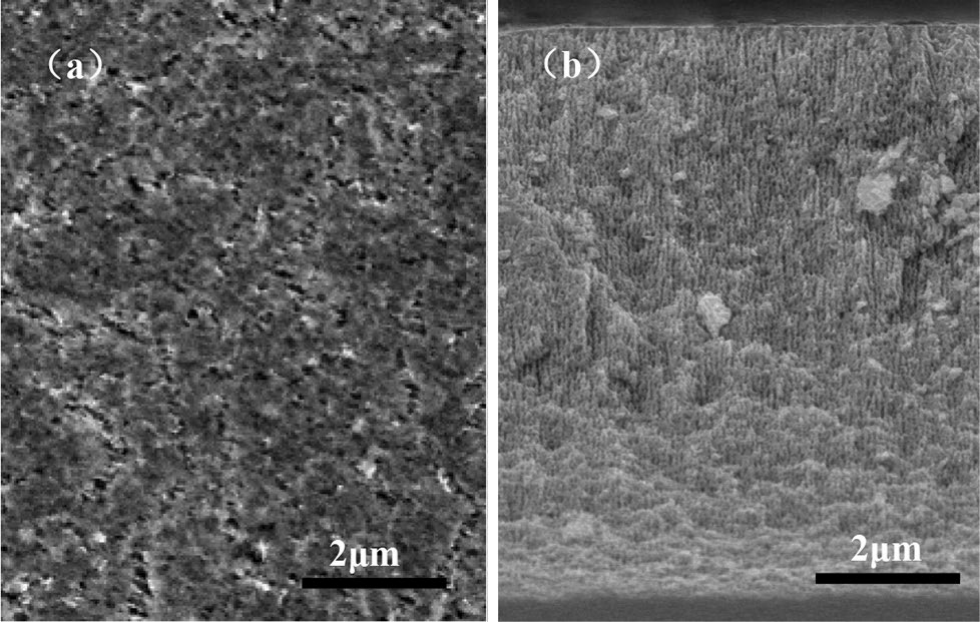
The etched InP prepared at 5 V for 2 min in 0.5 M HCl solution for (**a**) top-view SEM image and (**b**) cross-sectional SEM image.

Figure 1a,b presents the top-view and cross-sectional SEM image of the nanoporous InP etched at 5 V for 2 min in 0.5 M HCl aqueous solution. A large number of nanopore nuclei can form on the surface (Fig. 1a) and vertically aligned nanopores are observed in the cross-section SEM image (Fig. 1b). The etching depth is ~ 8 μm, and the etching rate is ~ 66.7 nm s^−1^. Based on previous report^8^, the electrochemical reaction of InP is as follows:

1 $$ {\text{InP}} + {\text{H}}_{2} {\text{O}} + {\text{h}}^{ + } \to {\text{In}}_{2} {\text{O}}_{3} + {\text{In}}({\text{PO}}_{3} )_{3} + {\text{H}}^{ + } $$

In addition, In_2_O_3_ are soluble in HCl, demonstrating a different reaction as follows:

2 $$ {\text{In}}_{2} {\text{O}}_{3} + {\text{H}}^{ + } \to {\text{In}}^{3 + } + {\text{H}}_{2} {\text{O}} $$

**Figure 2:**
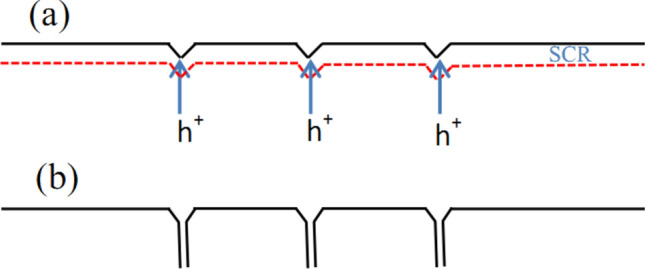
Schematic diagram of etching process. (**a**) Nanopores begin at pits of the InP surface. (**b**) Vertically aligned nanopores are formed in the InP.

To study the formation mechanism of nanopores in the InP, schematic diagrams are displayed in Fig. 2. Firstly, nanopores are formed at the pits of the InP surface as shown in Fig. 2a. It is reported that pits can be prepared via defect-related electrochemical etching^14,17^. Due to the interface curvature effects and the high electric field at the tips^18^, the generated holes results in the fact that nanopores are formed. When the space charge region (SCR) of neighboring nanopores overlap, the holes (h^+^) can be only generated along the vertical direction, and they result in the formation of vertically aligned nanopores as shown in Fig. 2b.

**Figure 3:**
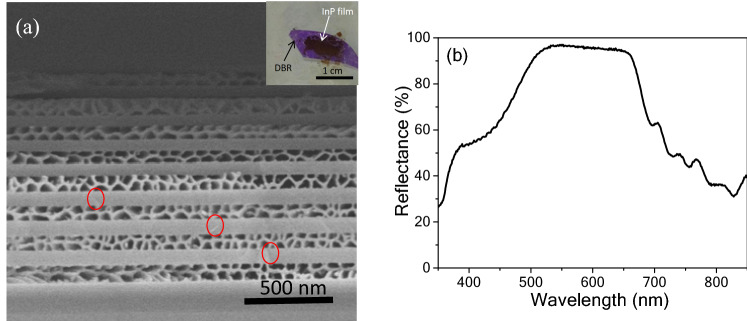
(**a**) Cross-sectional SEM image of MP-GaN DBR (The insert is the photograph of the nanoporous InP membrane transferred to an MP-GaN DBR), and (**b**) reflectance spectra of the bottom MP-GaN DBR.

Porosity can increase with the bias voltage rising^3,19,20^, i.e., the lift-off nanoporous InP thin films can be prepared via a variable voltage method. The insert of Fig. 3a shows the transferred nanoporous InP thin film. The voltage is first set to be 5 V for 2 min, and then it increases instantaneously to 10 V for 40 s. Afterwards, the self-supporting nanoporous InP thin film is transferred to the MP-GaN DBR substrate (Fig. 3a, (insert)). The MP-GaN DBR is prepared via an electrochemical etching. Figure 3a shows the cross-sectional SEM image of MP-GaN DBR obtained at 13 V for 15 min in NaNO_3_ solution via an electrochemical etching. The sparse pores (marked by red solid circles) appear in the undoped GaN layers, which exhibits the vertical etching due to the presence of defect^21^. However, n-GaN layers show high porosity, which shows the lateral etching owing to the restriction of neighboring undoped GaN layers.

Figure 3b shows the reflectivity spectrum of the MP-GaN DBR. The DBR shows a wide stopband (~ 140 nm) with high reflectivity (~ 96%) across the wafer. The same reflection spectra are got at the different regions of the DBR, indicating a uniform electrochemical etching. Particularly, the width of the stopband is larger than the reported value (~ 80 nm)^22^.

**Figure 4:**
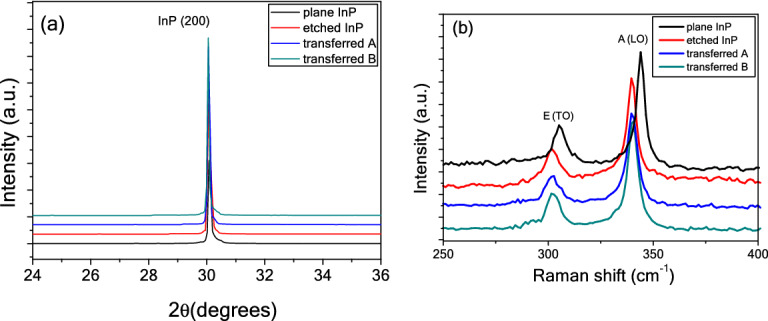
Four samples for (**a**) XRD patterns, (**b**) surface Raman spectra.

Figure 4a presents the XRD patterns of the bulk InP, etched InP, and two transferred nanoporous InP samples. Compared to bulk InP, the etched and transferred InP samples show stronger diffraction peaks, which is at least partially attributed to the increased the physical surface roughening. In addition, there are no significant shifts in the peak positions of InP (200) in the XRD patterns, i.e., change of residual stress in the etched layer is not obvious. The result is different from the formation of porous Si with high stress under a control current^23^, indicating the electrochemical etching under constant voltage is suitable for the preparation of nanoporous InP without the stress.

Figure 4b shows the surface Raman spectra of the four samples. The etched InP and transferred nanoporous InP samples show similar E (TO) peak positions (301 cm^−1^) and A (LO) peak positions (341 cm^−1^), while bulk InP presents those of 304 cm^−1^ and 345 cm^−1^, respectively. The band shifting to lower frequencies can be attributable to quantum confinement effect of phonon modes^24^. Compared to the bulk InP, the etched InP and transferred nanoporous InP samples also show symmetric Raman peaks and there is no broadening full width at half maximum (FWHM) of E (TO) modes and A (LO) modes, meaning the etched InP and transferred nanoporous InP samples still have good crystalline quality.

**Figure 5:**
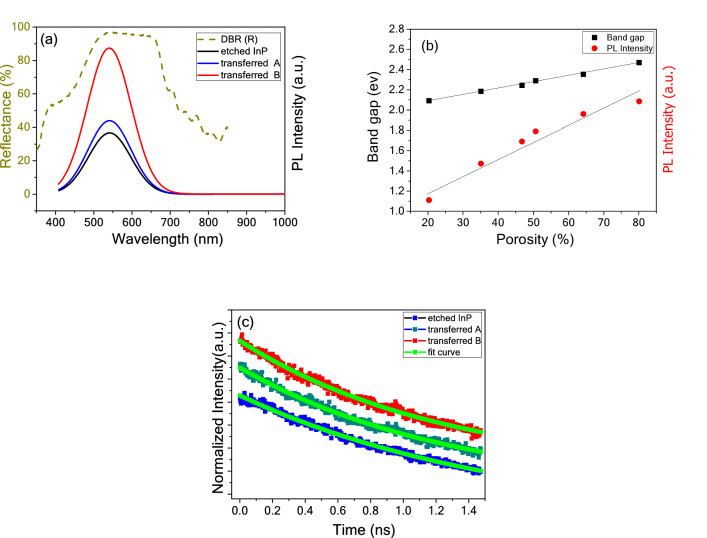
(**a**) PL spectra (solid line) of three nanoporous InP samples and reflectance spectra of DBR (dotted line), (**b**) band gap energy and PL Intensity vs. porosity with different etching voltage, and (**c**) normalized luminescence decay of three nanoporous InP samples.

Figure 5a exhibits the PL spectra of the three nanoporous samples using the exciting wavelength of 405 nm at 300 K. Compared to PL peak of bulk InP, those of the etched InP samples shift to the shorter wavelength (541.2 nm), which may be due to the quantum size effect of the nanoporous InP structure. The bandgap is inversely proportional to the wavelength of the PL peak. In another word, bandgap can widen as the crystalline size decreases, which is in accordance with previous reports on InP quantum dots and nanocrystals^25,26^, as well as porous Si^27,28^. Compared to the nanoporous InP which is not separated from the bulk InP, the nanoporous InP membrane transferred onto quartz substrate shows a slight enhancement in PL emission at 541.2 nm, due to the fact that cracks deriving from the transfer process leads to an increased light extracting area. More interestingly, the nanoporous InP membrane transferred onto MP-GaN DBR substrate shows a twofold enhancement in PL emission compared to the membrane transferred to quartz substrate. Since the MP-GaN DBR shows no PL peak in the experiment using the exciting wavelength of 405 nm, the twofold enhancement of PL intensity can be mainly attributable to the high light reflection effect of the bottom MP-GaN DBR^15^. In addition, Fig. 5a shows that the full width at half maximum (FWHM) of PL peak changes slightly after the transfer process, which is due to the cracks deriving from the transfer process.

As the etching voltage increases, the pore wall becomes thinner, and the porosity rises^20^. The bandgap (E_g_) can be calculated based on the equation (${E}_{g}=1239.5/\lambda$, λ is the PL peak position). Figure 5b shows the bandgap energy (E_g_) and PL intensity vs. porosity with different etching voltage. There is a proportional relationship between E_g_ and porosity, as well as PL intensity and porosity. The E_g_ and PL intensity of the sample increase as the porosity rises, which is due to the fact that the structure size (pore wall) of the nanoporous InP layer decreases markedly with increasing porosity. In short, both the E_g_ and PL intensity increase as the structure size decreases, proving that the visible PL of the nanoporous InP is caused by the quantum size effect.

Figure 5c exhibits the normalized luminescence decay of three samples at 541.2 nm at 300 K. Through a curve fitting via the double exponential decay function^29,30^, the etched InP and the transferred nanoporous samples show the same PL lifetime, meaning the lift-off and transfer process leads to negligible damage in crystalline quality.

## Conclusions

Both the lift-off nanoporous InP membranes and MP-GaN DBR are obtained by an electrochemical etching. The nanoporous InP samples show significant blue-shifts of PL emission compared to the bulk InP due to the fact that bandgap can widen as the crystalline size decreases. Additionally, the nanoporous InP membrane transferred onto DBR shows the largest PL intensity among the samples, presumably resulting from the high light reflection effect of the bottom MP-GaN DBR.

## Methods

### Sample fabrication

Electrochemical etching experiments are carried out in a two-electrode electrochemical cell with an n-type InP (100) wafer as the anode and a platinum (Pt) wire as the cathode^9^. The 450-μm-thick InP (100) wafer shows a doping density (*N*_*D*_) of $1\sim 4\times {10}^{18} {\mathrm{cm}}^{-3}$. The etching experiment was conducted in a constant voltage mode which is controlled by a source meter (GWINSTEK PPE-3323) under room light. Cl ions can be used as a catalyst in the etching process^8^, so the 0.5 M HCl aqueous solution was used as etching electrolyte. In addition, the lift-off nanoporous InP membranes are prepared by a variable voltage etching method, and then they are transferred to MP-GaN DBR or quartz substrates. The DBR is prepared by a two-electrode electrochemical cell with a GaN periodic structure as the anode and a Pt wire as the cathode. The GaN periodic structure consisting of n-type GaN (100-nm-thick; *N*_*D*_ = 2.5 × 10^19^ cm^−3^) and undoped GaN (50-nm-thick; *N*_*D*_ = 5 × 10^15^ cm^−3^) is etched at 13 V for 15 min in 0.3 M NaNO_3_ solution.

### Measurements

The morphology of the sample is studied by scanning electron microsopy (SEM) (JEOL JSM-6700F). Structure and optical characteristics of the samples are measured with D8 Advance x-ray diffractometer (XRD), double-beam UV–Vis–NIR spectrophotometer (TU-1901), Raman spectroscopy with an exciting wavelength at 632.8 nm and PL spectroscopy using 405 nm as the exciting wavelength, respectively.
